# A Case of Primary Tuberculous Parotitis Mimicking Parotid Neoplasm: A Rare Clinical Entity

**DOI:** 10.7759/cureus.58217

**Published:** 2024-04-13

**Authors:** Ramchandar Ramanan, Meenakshi Yeola, Nareshkumar P, Arundhathi S, Kheerthana Uma Maheswaran

**Affiliations:** 1 Surgery, Jawaharlal Institute of Postgraduate Medical Education & Research, Puducherry, IND; 2 General Surgery, All India Institute of Medical Sciences, Mangalagiri, Mangalagiri, IND; 3 Pathology, All India Institute of Medical Sciences, Mangalagiri, Mangalagiri, IND; 4 Otolaryngology - Head and Neck Surgery, SRM Medical College Hospital and Research Centre, Puducherry, IND

**Keywords:** extrapulmonary tuberculosis, parotid mass, parotitis, parotid neoplasm, primary tuberculous parotitis

## Abstract

Primary tuberculous parotitis is an extremely rare entity presenting with nonspecific symptoms, variable clinical signs, and imaging features mimicking parotid neoplasm. It is a clinical and diagnostic challenge, and a confirmed histological diagnosis would indicate nonoperative management, thus avoiding unwarranted surgery and associated morbidity. Tuberculosis of the salivary gland is a relatively rare extrapulmonary manifestation of tuberculosis, with the incidence of tuberculous parotitis being 2%-9%. The prevalence of disseminated tuberculosis has increased in recent times because of the use of immunosuppressive therapy for organ transplantation and chemotherapy. However, the incidence of concurrent pulmonary tuberculosis in patients with tuberculous parotitis is a rarer scenario. Fine-needle aspiration cytology (FNAC) can confirm the diagnosis of tuberculous parotitis with a high sensitivity (84%-100%) and specificity (94%-100%). The utility of FNAC is also enhanced as the aspirate can be utilized for cartridge-based nucleic acid amplification test (CBNAAT) testing for mycobacterium and drug sensitivity testing, thereby further increasing its sensitivity and specificity. This translates to a lesser chance of unnecessary surgical intervention and the potential surgical morbidity. Here, we report a case of parotid swelling in a 72-year-old male, with no evidence of any pulmonary or systemic tuberculosis, with clinical and imaging features suggestive of parotid neoplasm but diagnosed as tubercular parotitis on FNAC. He was started on antitubercular therapy, which resulted in the progressive diminution of the size of the lesion. Primary tuberculous parotitis should be considered a possibility while managing the parotid neoplasm.

## Introduction

The overall incidence of extrapulmonary tuberculosis is found to be around 15-20%. Tuberculosis of the parotid gland is a very rare form of extrapulmonary tuberculosis even in countries such as India where tuberculosis is still considered an overburdening disease. Clinically, it presents as a slow-growing localized mass, indistinguishable from a parotid neoplasm, making it a diagnostic dilemma and an extremely difficult condition to clinch the early diagnosis of tuberculous parotitis. Hence, a high index of clinical suspicion is required in diagnosis of the tuberculous parotitis as delayed diagnosis or misdiagnosis would entirely result in unwarranted surgery and untoward complications in the patient. The identification of mycobacterium complex is an important step in initiating antitubercular therapy, and fine-needle aspiration cytology (FNAC) seems to be the less invasive technique.

## Case presentation

**Figure 1:**
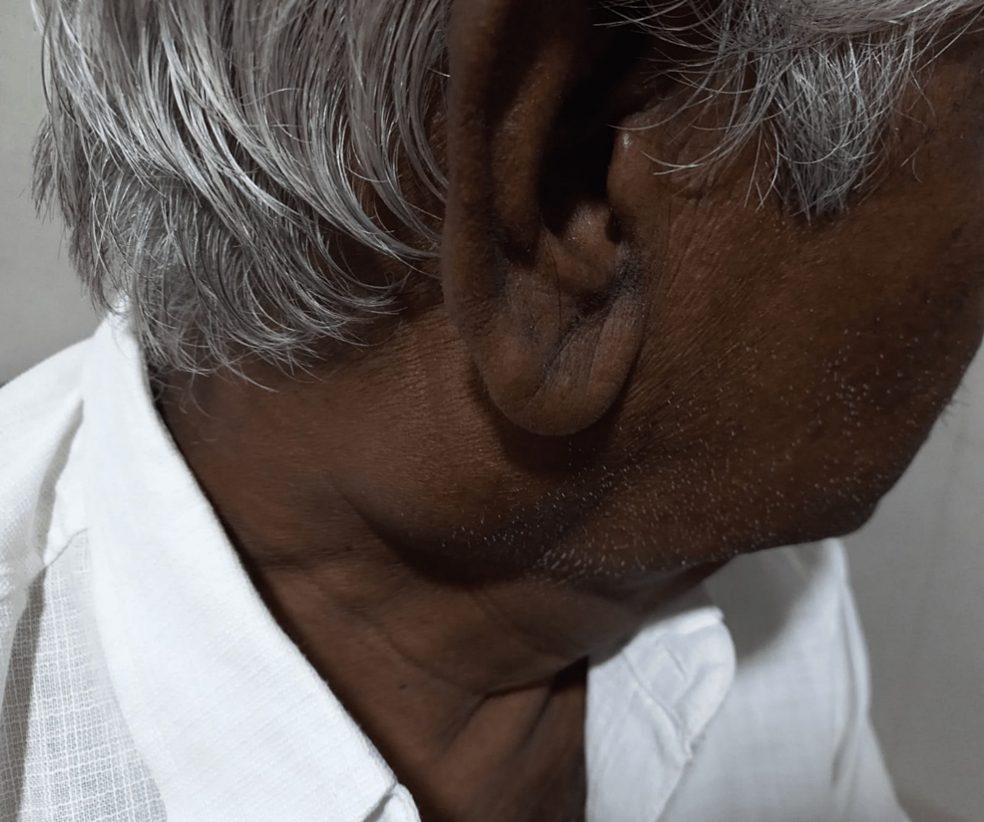
Patient with a right parotid enlargement

**Figure 2:**
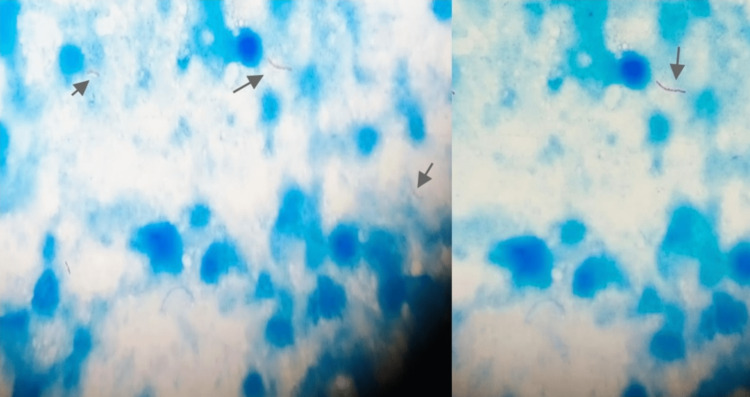
FNAC aspirate showing a Ziehl–Neelsen stain positive for AFB AFB: acid-fast bacilli

**Figure 3:**
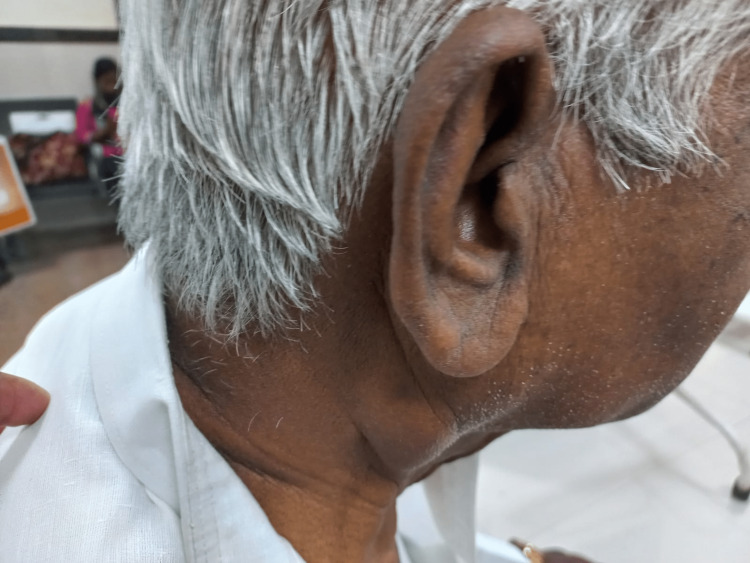
Showing diminution in the size of the parotid after two months of ATT ATT: anti-tuberculosis treatment

A 72-year-old gentleman presented to the OPD with complaints of swelling over the right parotid region for the past four years, which was insidious in onset, gradually progressive in size, and not associated with any history of fever, weight loss, night sweats, chills and rigor, and previous surgery. No relevant past medical history was present. A physical examination revealed diffuse swelling of the right parotid gland, which was non-tender, firm in consistency, and without any skin changes (Figure 1). Laboratory investigations were normal, except for the elevation of the C-reactive protein of 14 mg/dL. The chest X-ray was normal. An ultrasound of the neck revealed an ill-defined hypoechoic lesion measuring 4.4x3.0 cm, likely arising from the right parotid gland showing exophytic extension inferiorly with few adjacent reactive lymph nodes, surrounding fat stranding and minimal internal vascularity noted. The diagnostic possibility of infective or neoplastic etiology arising from the parotid gland could not be ruled out at that time. An FNAC of the swelling was performed, which was found to be sparsely cellular and consisting of degenerative inflammatory cells against a necrotic background, and the Ziehl-Neelsen stain (ZN) stain for the acid-fast bacilli (AFB) was positive (Figure 2). The USG-guided FNAC aspirate was sent for cartridge-based nucleic acid amplification test (CBNAAT) and reported to be positive for *Mycobacterium tuberculosis* with sensitivity to rifampicin. The patient was started on anti-tuberculosis drugs for six months and is under regular follow-up. After 10 weeks of initiation of anti-tuberculosis treatment (ATT), there was clinical evidence of a decrease in the size of the swelling (Figure 3).

## Discussion

Tuberculosis of the salivary glands is an extremely rare extrapulmonary manifestation, and the incidence of tuberculosis of the parotid gland is around 2%-9% [1]. Tuberculous parotitis more commonly presents as a unilateral disease, and its incidence is around 5% [2]. The incidence of concurrent pulmonary tuberculosis with parotid involvement is considered rare, which is around 25%, and makes arriving at a prompt diagnosis even more difficult. Moreover, a high index of suspicion is needed to diagnose tuberculous parotitis. Similarly, in our case, there was no evidence of any active pulmonary tuberculosis: the chest X-ray and sputum AFB were negative. Tuberculosis of the parotid may present with varied manifestations such as acute and chronic sialadenitis. Acute forms may present as abscesses, and chronic forms may present as long-standing painless swelling with or without lymph node involvement, which can mimic parotid neoplasms similar to our patient. The spread of mycobacterium to the parotid gland occurs when an infective focus in the oral cavity liberates bacilli that ascend to the gland via ducts or pass to their associated lymph nodes via lymphatic drainage [3]. No evidence of hematogenous spread was noted. Imaging techniques are of less diagnostic value [4]. In most cases, extrapulmonary tuberculosis is primarily a pauci bacillary disease, making the conventional method of smear microscopy less sensitive, and, in most cases, invasive diagnostic procedures are routinely utilized. FNAC is a less invasive procedure and a more reliable technique in the diagnosis of tuberculous parotitis with a high sensitivity (84%-100%) and specificity (94%-100%) [5]. Hence, FNAC is considered an initial investigation of choice in any parotid mass, particularly for tuberculosis parotitis, as the fine-needle aspirate is utilized for CBNAAT, and drug-sensitivity testing of mycobacterium further increases the sensitivity of FNAC [6]. Two different histopathological forms of parotid tuberculosis have been reported as localized and diffuse types. In the localized type, initially, there is involvement of pericapsular or intracapsular lymph nodes; conversely, in the diffuse type, there is predominant and diffuse involvement of salivary gland parenchyma initially [7]. Preoperative diagnosis would prevent unwarranted surgery and medical treatment with antitubercular drugs should be continued for six to nine months [8].

## Conclusions

Early diagnosis is pivotal in the management of tuberculous parotitis to prevent surgery, and its complications as the starting of ATT promptly cures the disease completely. Hence tuberculous parotitis should be kept in mind as a differential diagnosis in patients with long-standing nonspecific parotid swelling because the entire line of management differs by a large extent, thereby preventing unwarranted surgery and its complications.
